# Formation, Toxicity, and Analytical Techniques for Small-Molecule α-Dicarbonyl Compounds in Foods

**DOI:** 10.3390/foods15142566

**Published:** 2026-07-21

**Authors:** Ningbo Wan, Yao Wang, Lijuan Wang, Hongyun Wang, Zhaozhou Li, Lei Hua, Huawei Niu, Xiujin Chen, Jianrui Sun

**Affiliations:** 1 College of Food and Bioengineering, Henan University of Science and Technology, Luoyang 471023, China; ningbo_wan@163.com (N.W.); wangyao@haust.edu.cn (Y.W.); wanghy040208@163.com (H.W.); chenxiujin9610@126.com (X.C.); dasheng@haust.edu.cn (J.S.); 2 Liaoning Key Laboratory for Mass Spectrometry Technology and Instrumentation, Dalian Institute of Chemical Physics, Chinese Academy of Sciences, Dalian 116023, China; 3 College of Basic Medical Science, Ningxia Medical University, Yinchuan 750004, China

**Keywords:** small-molecule α-DCs, high reactivity, the Maillard reaction, caramelization, chromatography, chromatography–mass spectrometry

## Abstract

Small-molecule α-dicarbonyl compounds (α-DCs), including glyoxal, methylglyoxal and diacetyl, are electrophilic compounds characterized by two adjacent carbonyls. These compounds ubiquitously occur in various foods and food–medicine homologous herbs, generated via the Maillard reaction, caramelization, lipid peroxidation, and enzymatic reactions during thermal treatment and storage. Upon oral intake, small-molecule α-DCs are rapidly absorbed into systemic circulation, triggering protein and DNA damage, as well as inflammation. They also serve as important precursors to derive other hazards, such as advanced glycosylation end products possessing carcinogenic and genotoxic properties. Small-molecule α-DCs and their derived harmful products accelerate the progression of multiple metabolic diseases, e.g., cancer and diabetes. However, their pathological processes remain poorly elucidated, necessitating highly sensitive and accurate analytical methods. This review also systematically summarizes and discusses the current analytical techniques targeting small-molecule α-DCs. Chromatography and chromatography–mass spectrometry are still frequently used techniques. Given the polarity and weak ultraviolet absorption of small-molecule α-DCs, tedious pretreatment is necessary yet time-consuming. Novel rapid detection techniques such as mass spectrometry probes and direct ionization mass spectrometry have been proposed in recent years. Even so, developing rapid, eco-friendly, highly sensitive and accurate analytical methods remains a key priority for future research.

## 1. Introduction

Alpha-dicarbonyl compounds (α-DCs) represent a category of electrophilically active substances containing two adjacent carbonyl groups. In recent years, small-molecule α-DCs, primarily comprising glyoxal (GO), methylglyoxal (MGO), and diacetyl (DA), have garnered increasing research interest owing to their severe toxicity, high chemical reactivity, wide occurrence in food, and difficulty in detection [1,2,3]. People are exposed to small-molecule α-DCs mainly through their diets. During thermal processing or prolonged storage of food products, small-molecule α-DCs can be formed via the Maillard reaction, autoxidation of carbohydrates, lipid peroxidation, microbial enzymatic reactions, and so on [1]. According to their formation pathways, small-molecule α-DCs ubiquitously exist in various foods and Chinese herbal medicines [2,4,5,6,7]. Concentrations of small-molecule α-DCs are high in wines, soy sauce, honey, dried fruits, and fruit-based Chinese herbal medicines, which can be attributed to microbial metabolism from the fermentation process, high content of reducing sugars and fats, and intense heat treatment [8,9,10]. For instance, the highest concentrations of GO, MGO, and DA are 60.48 mg kg^−1^ in plum wine, 736 mg kg^−1^ in manuka honey, and 55.06 mg kg^−1^ in raspberry wine, respectively [1,11]. The main factors affecting the levels of small-molecule α-DCs are the type of sugars and the extent of heat impact during manufacturing; other processes like the reduction of water, ripening, fermentation, and storage also promote the formation of small-molecule α-DCs [1,12,13]. Moreover, geographical regions and vegetational diversity of the environment also affect the contents of small-molecule α-DCs. For example, the content of MGO in manuka honey from China ranges from 124.5 to 530 mg kg^−1^ [14], whereas the same honey from the Netherlands demonstrates an MGO content ranging from 2.5 to 736 mg kg^−1^ [1].

Dietary exposure to small-molecule α-DCs is of considerable concern, as these compounds are rapidly absorbed into the systemic circulation via the gastrointestinal tract. The imbalance between small-molecule α-DC formation and degradation gives rise to the phenomenon known as “dicarbonyl stress”, which directly leads to the accelerated oxidation of carbohydrates and lipids, particularly an increase in reactive oxygen species homeostasis levels and cellular metabolic disorders [15,16,17]. A large number of studies indicate that small-molecule α-DCs, particularly GO and MGO, are carcinogenic, genotoxic, and neurotoxic and are closely linked to the onset and progression of numerous human diseases, including cancer, diabetes mellitus, Alzheimer’s disease, etc. [18,19]. For instance, GO and MGO are key factors for diabetes and its complications. Research showed that the levels of GO and MGO in the blood of diabetic patients were 0.15~0.26 μg mL^−1^ and 0.16~0.27 μg mL^−1^, respectively, which were 2.3–10-fold higher than those observed in healthy individuals [19]. DA has been demonstrated to induce necrosis in the respiratory epithelium and drive progressive fibrotic obliteration of bronchioles [18].

**Figure 1:**
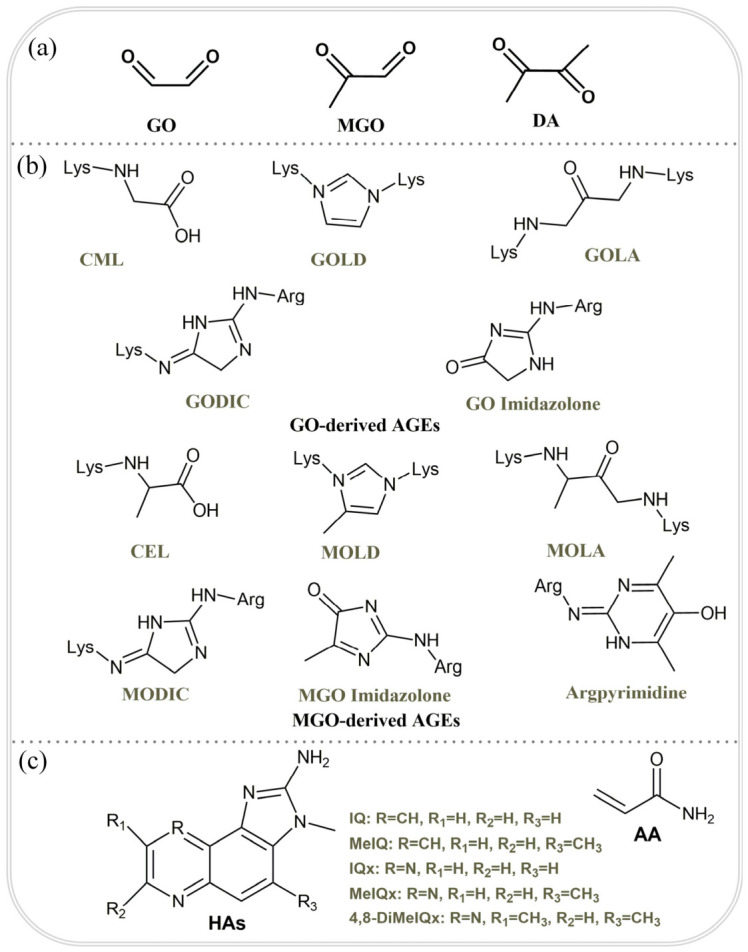
Structures of small-molecule α-DCs (**a**), small-molecule α-DC-derived AGEs (**b**), AA and HAs (**c**).

Small-molecule α-DCs can also act as important intermediates and precursors by inducing other toxic compounds, such as acrylamide (AA) [20], advanced glycation end products (AGEs) [21], and heterocyclic amines (HAs) [22] in foods [16]. The structures of small-molecule α-DCs and their derived toxic compounds are shown in Figure 1. AA is neurotoxic and genotoxic [20] and was classified as a Group 2A potential carcinogenic substance to humans by the International Agency for Research on Cancer (IARC) as early as 1994. Studies have reported that GO generated AA at a 235-fold higher efficiency than reducing sugars [23]. Small-molecule α-DCs exhibit high chemical reactivity toward the lysine and arginine side chains of proteins, resulting in a variety of stable protein adducts, which are summarized as AGEs [24]. AGEs are mutagenic and can cause harm to human health. The molecular structures of AGEs mainly derived from GO or MGO are elucidated as follows: imidazolium-type derivatives are glyoxal lysine dimer (GOLD) and methylglyoxal lysine dimer (MOLD), while amide-type AGEs are glyoxal lysine amide (GOLA) and methylglyoxal lysine amide (MOLA). It has been shown that HAs can be generated by a series of reactions of small-molecule α-DCs; typical representatives reported in the literature include 2-amino-3-methylimidazo [4,5-ƒ] quinoline (IQ), 2-amino-3,4-dimethylimidazo [4,5-ƒ] quinoline (MeIQ), 2-amino-3,8-dimethylimidazo [4,5-ƒ] quinoxaline (MeIQx), 2-amino-3-methylimidazo [4,5-ƒ] quinoxaline (IQx) and 2-amino-3,4,8-trimethylimidazo [4,5-ƒ] quinoxaline (4,8-DiMeIQx) [25,26]. Among these, IQ has been classified as a Group 2A probable carcinogen and the others as Group 2B possible carcinogens by the IARC [27]. Epidemiological studies have shown that the hazards induced by small-molecule α-DCs would elevate the risk of cancer, cardiovascular disease, Parkinson’s disease, and other chronic illnesses [28,29,30].

**Figure 2:**
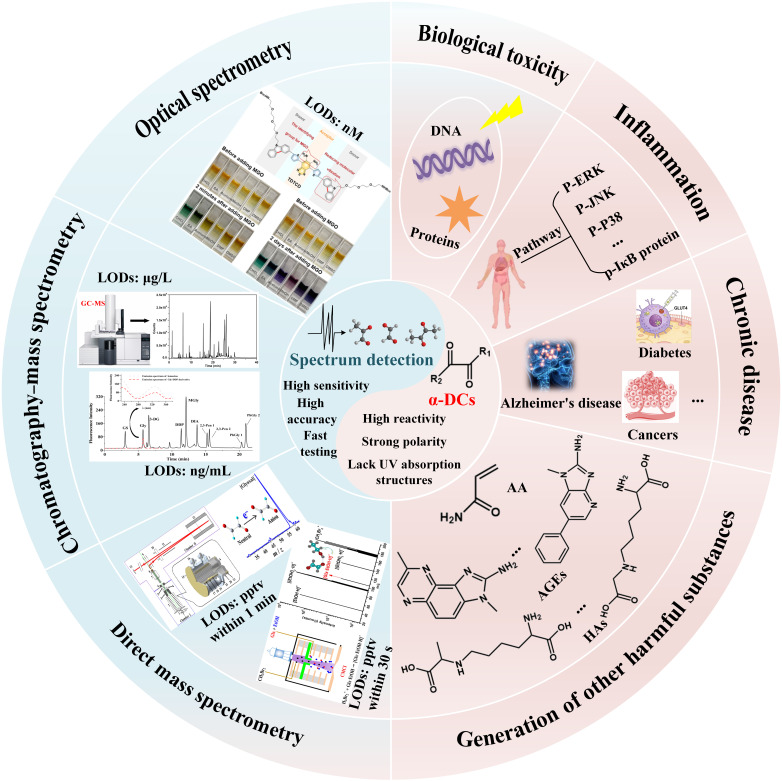
Hazardous effects and detection techniques of small-molecule α-DCs, including optical spectrometry, figure adapted from ref. [31], with permission from the Royal Society of Chemistry, copyright 2026; chromatography–mass spectrometry, figure adapted from ref. [32], with permission from the Royal Society of Chemistry, copyright 2026; and direct mass spectrometry, figures adapted from ref. [33], with permission from the American Chemical Society, copyright 2026, ref. [34], with permission from Elsevier, copyright 2026, and ref. [35], with permission from Peking University, copyright 2026.

As shown in Figure 2, due to the serious health risks posed by small-molecule α-DCs, as well as their widespread existence in various foods and Chinese herbal medicines, the investigation into techniques for their control and detection has long remained a research focus in food chemistry and food safety. However, the high chemical reactivity and strong polarity of small-molecule α-DCs make it challenging to perform accurate quantitative analyses. Reliable analytical methods are essential for estimating their safety and preventing excessive exposure to small-molecule α-DCs. In this respect, this review retrieved relevant publications from the Web of Science (WOS) database using dicarbonyl compounds as the core search term. Literature screening was performed based on research themes concerning the formation mechanisms, toxicity or hazards, and analytical techniques of glyoxal (GO), methylglyoxal (MGO), and diacetyl (DA) in foods. Approximately 85% of the eligible studies were published between 2014 and 2026, which provide key evidence for elucidating the generation and transformation patterns of small-molecule α-DCs during food processing and reducing their adverse impacts on human health.

## 2. The Formation of Small-Molecule α-Dicarbonyl Compounds

Small-molecule α-DCs like GO, MGO, and DA are formed during the thermal processing and storage of foods high in carbohydrates, proteins, or lipids, especially sugar-rich foods. Excessive dietary exposure to these small-molecule α-DCs will bring serious health risks. Therefore, elucidating the formation mechanism of these small-molecule α-DCs throughout food thermal treatment is essential to regulate their contents in food products. The main mechanisms of small-molecule α-DC formation encompass both non-enzymatic and enzymatic processes. Non-enzymatic routes include the Maillard reaction, caramelization, and lipid peroxidation, while enzymatic mechanisms involve specific catalytic reactions mediated by metabolic enzymes.

### 2.1. The Maillard Reaction

**Figure 3:**
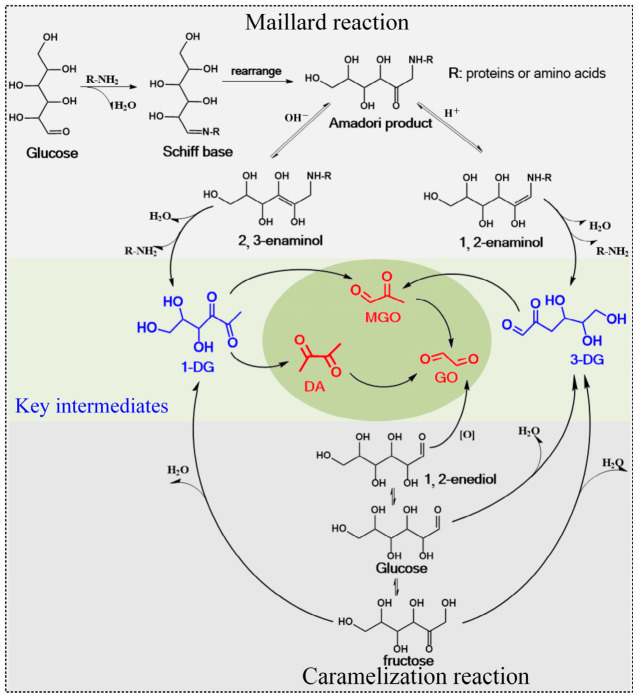
Formation of small-molecule α-DCs by the Maillard reaction and caramelization reaction; this figure was redrawn from ref. [2], with permission from John Wiley and Sons, copyright 2026, and ref. [27], with permission from the American Chemical Society, copyright 2026.

The most important mechanism of small-molecule α-DC formation during food heat processing is the Maillard reaction, a thermally driven reaction between carbohydrates and amino acids or proteins [2,36]. As shown in Figure 3, the Maillard reaction is initiated via Schiff base formation, which arises from the dehydration reaction between a reactive carbonyl compound (e.g., glucose) and a primary amine (e.g., proteins, peptide, or free amino acids); the unstable Schiff base rearranges to form Amadori products, which are stable keto-amine intermediates generated in the early Maillard reaction, chemically named 1-amino-1-deoxy-2-ketoses. Amadori products can undergo two different reaction routes to form small-molecule α-DCs. Under acidic conditions, the initial isomerization of Amadori products yields a 1,2-enaminol intermediate, which subsequently undergoes dehydration to generate 3-deoxyglucosone (3-DG). The 3-DG then undergoes further cleavage, resulting in the formation of MGO and GO. In another pathway, the Amadori product isomerizes to form 2,3-enaminol under alkaline conditions, which can subsequently dehydrate to yield 1-deoxyglucosone (1-DG) and then ultimately degrade into DA and GO. In addition to acidity and alkalinity, the generation of small-molecule α-DCs via the Maillard reaction is also influenced by the type of reactant substrates, moisture, processing method, heating temperature, and duration [12]. Çatak et al. [13] observed that fructose exhibited a positive correlation with the formation of GO and MGO, whereas sucrose presented a negative relationship. The reason may stem from the structural characteristics of sucrose: as a disaccharide composed of glucose and fructose units linked by a glycosidic bond, its molecular structure imposes steric and electronic constraints that collectively suppress its overall chemical reactivity.

### 2.2. Caramelization

Another important pathway responsible for generating small-molecule α-DCs is caramelization (Figure 3). Caramelization is a non-enzymatic browning reaction that typically takes place in foods rich in reducing sugars, including honey, dried fruits, fruit-based Chinese herbal medicines, and so on [27]. Polysaccharides, such as maltose and starch, contribute to caramelization by undergoing hydrolysis into their corresponding monosaccharides, thereby promoting the generation of small-molecule α-DCs. The mechanisms underlying α-DC formation through caramelization are illustrated in Figure 3. Dehydration and cleavage are the core reactions for the formation of small-molecule α-DCs. Under elevated thermal conditions, monosaccharides (e.g., glucose or fructose) undergo isomerization via 1,2-enolization to generate unstable enol intermediates. These enols subsequently lose water molecules through dehydration, yielding highly reactive species. The latter participate in a cascade of sequential reactions, including further dehydration, β-elimination, bond cleavage, retro-aldol reactions, aldol condensation, and radical-mediated transformations, to ultimately produce small-molecule α-DCs. It is worth noting that both the Maillard reaction and caramelization can form the intermediates 1-DG and 3-DG, which proceed to generate small-molecule α-DCs in parallel and interact with each other during high-temperature processing. When amino-containing substances are absent, the caramelization reaction dominates; in contrast, the Maillard reaction constitutes the primary pathway for the generation of small-molecule α-DCs.

### 2.3. Lipid Peroxidation

Lipid peroxidation can be primarily defined as the oxidative decomposition of polyunsaturated fatty acids (PUFAs) triggered by external stimuli such as light, high-energy radiation, and heat [2,37]. In contrast to the Maillard and caramelization reactions, small-molecule α-DCs produced by lipid peroxidation account for a relatively minor fraction. The predominant pathway for generating small-molecule α-DCs via lipid peroxidation involves the oxidative degradation of PUFAs during food thermal processing or storage. This process follows a free-radical chain mechanism, comprising initiation, propagation, and termination stages, which subsequently yield a mixture of lipid hydroperoxides (LOOH). Under the catalysis of heat, light, or metal ions, unstable LOOH undergoes O-O bond cleavage, yielding highly reactive alkoxy radicals (LO·) and hydroxyl radicals (·OH). The alkoxy radical (LO·) is extremely unstable and degrades via β-cleavage or cyclization into small-molecule α-DCs [38]. For example, the contents of small-molecule α-DCs increased significantly when the temperature increased from 100 to 200 °C. The specific cleavage pathway and fatty acid species also affect the types of small-molecule α-DCs [39]. For example, the peroxidation of ω-3 fatty acids can produce GO and MGO. Initially, ω-3 fatty acids are converted into ω-7 hydroperoxide, which can undergo further hydroperoxidation to form 1,2- or 1,6-dihydroperoxides. 1,2-dihydroperoxide is a precursor to epoxy-dihydroperoxide, whose cleavage yields GO. Meanwhile, 1,6-dihydroperoxide decomposes to produce 6-hydroperoxy-2,4-heptadienal, which is subsequently converted to 4-oxo-2-pentenal via the loss of two carbon atoms, with a characteristic feature of 2,4-dienals. The latter is known as a precursor of MGO and GO [40].

### 2.4. Enzymatic Reactions

Small-molecule α-DCs can be formed through enzymatic reactions, especially during the fermentation of foods. For example, the microorganisms *Thermoanaerobacterium thermosaccharolyticum* [41], *Corynebacterium glutamicum* [42], and *Synechocystis* [43] were reported to ferment common sugars (glucose, lactose, galactose, and so on) to form dihydroxyacetone phosphate (DHAP) in the process of glycolysis. DHAP can be converted to MGO via the catalytic action of MGO synthase [44,45]. However, during thermal processing of food, enzymatic reactions do not directly generate significant amounts of small-molecule α-DCs. Instead, their primary role is to efficiently and selectively synthesize large pools of highly reactive precursors during the initial heating phase. In subsequent heating and thermal processing, these precursors, primarily specific aldehydes and quinones, act as “fuel” and undergo rapid non-enzymatic transformations (likely via the Maillard reaction, caramelization, or lipid peroxidation) to yield small-molecule α-DCs.

## 3. The Occurrence of Small-Molecule α-DCs in Foods

Based on their formation mechanisms, small-molecule α-DCs are widely present in diverse foods, including honey, beverages, bread, coffee, sauces, meat, dairy products, and oils (Table 1). The composition and concentrations of small-molecule α-DCs in foods are influenced by processing approaches, ingredient formulations, storage environments, and various other factors [1,46,47].

As displayed in Table 1, GO, MGO, and DA concentrations are all relatively high in wine, especially fruit wine. This phenomenon is attributed to the combined effects of microbial metabolism, the Maillard reaction at high temperatures, and chemical reactions that occur during prolonged aging and storage. For example, in raspberry wine, the contents of GO, MGO, and DA are 44.27 mg kg^−1^, 40.42 mg kg^−1^, and 55.06 mg kg^−1^, respectively. Dried fruits also have high concentrations of small-molecule α-DCs, which may be due to their high content of reducing sugar, thereby resulting in non-enzymatic browning reactions during thermal processing. Besides wines and dried fruits, honey, cookies, sauces, and seafood also contain relatively high levels of GO and MGO. The highest concentrations are found in honey; the main reason is that honey contains high levels of monosaccharides. Milk and milk products contain relatively high levels of DA. The elevated DA concentrations observed in these products are mainly attributed to the metabolic activities of lactic acid bacteria (LAB). Specifically, LAB facilitate citric acid metabolism and the formation of α-acetyl lactate, which subsequently undergoes decarboxylation to yield DA.

The widespread distribution of small-molecule α-DCs in a variety of foods and beverages has been well documented. However, their presence in Chinese herbs remains largely unexplored. Moreover, many phytomedicines require processing procedures (such as baking, frying, steaming, and boiling) to enhance their quality and therapeutic efficacy before use [68,69]. These procedures favor the occurrence of the Maillard reaction, the caramelization of sugars, or lipid peroxidation in Chinese herbs, leading to the formation of small-molecule α-DCs. Recently, some researchers have focused on the quantitative analysis of small-molecule α-DCs in Chinese herbs and medicine products. In 2019, Lee et al. [64] first analyzed small-molecule α-DCs in red ginseng, and they found that red ginseng products contained 0.19–4.27 mg kg^−1^ of GO, 1.37–4.80 mg kg^−1^ of MGO, and 0–0.83 mg kg^−1^ of DA. Subsequently, Tang et al. [12], Yang et al. [5], and Xu et al. [70] investigated α-DCs in other Chinese herbal medicines. They found a wide occurrence of small-molecule α-DCs, with GO up to 26.8 mg kg^−1^, MGO up to 55.50 mg kg^−1^, and DA up to 18.75 mg kg^−1^. These findings indicate that the contents of small-molecule α-DCs in Chinese herbs are generally comparable to those observed in various foods, implying that the small-molecule α-DCs in herbal medicines have potential risks to human health.

## 4. Toxicity of Small-Molecule α-DCs

Small-molecule α-DCs are generated in the process of heat treatment, fermentation, and long-term storage of various foods. Due to their high reactivity, small-molecule α-DCs can impair protein function through covalent modification, leading to carbonyl stress. Carbonyl stress results in elevated contents of reactive oxygen species and cellular metabolic dysfunction [71]. Small-molecule α-DCs can also induce DNA damage through oxidative modification of DNA bases, strand breaks, and cross-linking. These events can trigger mutations and genomic rearrangements, thereby facilitating the initiation and progression of cancer [17]. Furthermore, small-molecule α-DCs can activate several signaling pathways, such as P-ERK, P-JNK and NF-κB, to promote inflammatory responses [26,72]. Liu et al. [73] investigated the impact of GO on the NF-κB signaling pathway by measuring both p-IκB and IκB protein levels. They revealed that the p-IκB protein expression level was upregulated and the ratio of p-IκB/IκB was markedly elevated as GO concentrations increased from 29 to 116 mg L^−1^. These findings suggest that GO may trigger inflammatory injury by activating the NF-κB signaling pathway. Small-molecule α-DCs have also been strongly related to the progression of several chronic diseases. In a Drosophila model, a 1.5-fold elevation in MGO concentration relative to a control group induced core phenotypes of type 2 diabetes (T2D), including hyperglycemia, obesity, and increased insulin resistance [74]. In a follow-up study involving 1003 individuals with T2D, Hanssen et al. measured the plasma levels of MGO, GO, and 3-deoxyglucosone. Their results demonstrated that elevated plasma MGO and GO concentrations are correlated with increased cardiovascular disease-related mortality in patients with T2D [75]. DA has been demonstrated to induce necrosis in respiratory epithelial cells and trigger bronchiolar obliteration. Inhaling high concentrations of DA vapor can cause inflammatory responses and fibrosis in the small airways of the lungs, resulting in permanent breathing difficulties, which is a stark warning of DA’s high cytotoxicity [18].

Small-molecule α-DCs with a 1,2-dicarbonyl structure forming the active site can act as key intermediates in the derivation of other harmful substances. Small-molecule α-DCs can undergo non-enzymatic reactions with free amino groups on proteins, amino acids, and nucleic acids to form AGEs, which constitute the core indirect hazard mechanism of small-molecule α-DCs [21]. Lysine is the primary reactant substrate for the formation of AGEs due to its ε-NH_3_ group, which is highly nucleophilic and reactive. In the best understood example, GO reacts with lysine, leading to imidazolium cross-link glyoxal lysine dimer (GOLD) and N^ε^-(carboxymethyl)-lysine (CML), as well as the amide structures glyoxal lysine amide (GOLA), N^6^-glycolyl lysine (GALA), and amidine structure glyoxal lysine amidine (GLA). MGO can also react with lysine to form the structurally analogous substances methylglyoxal lysine dimer (MOLD), argpyrimidine, N^6^-carboxyethyl lysine (CEL), methylglyoxal lysine amide (MOLA), and N^6^-lactoyl lysine. Except for argpyrimidine, whose formation involves two molecules of MGO participating through glycation and aldol reactions [76], all other AGEs formed from MGO incorporate just a single MGO molecule. A large number of studies have confirmed that AGEs are highly hazardous chemicals that promote cell death and contribute to organ damage. AGEs are implicated in the progression of many intractable disorders, such as diabetes and atherosclerosis, as well as in pathologic changes in chronic degenerative diseases, such as Alzheimer’s disease, Parkinson’s disease, and alcoholic brain injury [77,78].

Small-molecule α-DCs can also promote the formation of AA and HAs in food products, which cause serious harm to human health. Small-molecule α-DCs react with asparagine to initiate a cascade of chemical reactions, ultimately generating AA, which is a neurotoxin and potential human carcinogen [79]. Moreover, small-molecule α-DCs are more effective than reducing sugars in AA formation. In a reaction model (120 °C, 10 min) consisting of 25 mM dicarbonyl compounds and asparagine, GO induced 256- and 363-fold higher levels of AA relative to glucose and fructose, respectively. In comparison, MGO and DA formed 2.4–3.4 times and 1.3–1.8 times higher AA concentrations, respectively, compared to model systems containing the two hexoses [23]. Thus, it is concluded that GO is the most important intermediate among the small-molecule α-DCs and plays a crucial role in AA formation. Additionally, small-molecule α-DCs can induce the generation of HAs. For example, the reaction between GO and primary Maillard reaction intermediates can generate pyridine and pyrazine radicals, which subsequently interact with creatinine and aldehydes to generate IQ, IQx, MeIQ, MeIQx, and 4,8-DiMeIQx [27]. MGO is also the key precursor for MeIQx formation [25]. HAs represent a group of carcinogenic aromatic compounds generated during the thermal treatment of protein-rich foods, especially meats. Epidemiological evidence has established that exposure to HAs can elevate the risk of developing colorectal cancer, esophageal cancer, Parkinson’s disease, and other diseases [80].

## 5. Detection Techniques for Small-Molecule α-DCs

Small-molecule α-DCs are widely present in various foods, beverages, and Chinese herbal medicines. While their association with the onset and progression of multiple diseases has been documented, the precise mechanisms underlying their pathogenic roles and contributions remain elusive. Research into the formation and transformation patterns of small-molecule α-DCs, as well as their pathogenic mechanisms, relies heavily on detection techniques. At present, various detection techniques have been developed, including optical spectroscopic techniques, mass spectrometry, electrochemical methods [81], and enzyme-linked immunoassays (ELISAs) [82]. Among these, chromatography and chromatography–mass spectrometry techniques are most commonly used, as shown in Table 2. Therefore, this review synthesizes current analytical strategies based on spectroscopy and mass spectrometry for quantifying small-molecule α-DCs, assesses their analytical performance, and highlights recent innovations and future directions in food safety and applications.

### 5.1. Optical Spectroscopy

Compounds interact with electromagnetic waves, absorbing or emitting light of specific energies to produce unique “fingerprint” spectra. The composition, structure, and concentration of the compounds can be inferred by analyzing these spectra. Many researchers have used optical spectroscopy techniques to analyze small-molecule α-DCs. Ultraviolet (UV) spectroscopy and fluorescence (FL) spectroscopy are the most commonly used techniques, followed by nuclear magnetic resonance (NMR), surface-enhanced Raman scattering (SERS), and infrared (IR) spectroscopy.

#### 5.1.1. Ultraviolet Spectroscopy

The detection principle of UV spectroscopy is as follows: Each α-DC possesses two carbonyl groups (C=O) containing conjugated π electrons that absorb UV radiation. By analyzing the wavelength and intensity of UV peaks, we can directly identify and characterize these organic molecules. UV spectroscopy has been used to analyze the behavior of small-molecule α-DCs in the UV region since the 1990s. Meller et al. [86] first employed a conventional UV spectroscopic technique (220~490 nm) to detect MGO and found that it exhibited two high cross-sectional values within the range of 370 to 450 nm. Subsequently, using high-resolution UV spectroscopy, Volkamer et al. [87] reported that the UV absorption cross-section values of GO were 10–30% higher at 250 nm and 526 nm than those reported in prior studies. These studies have provided valuable insights into analyzing the molecular structures of small-molecule α-DCs through UV spectroscopy.

The trace levels of small-molecule α-DCs in foods, combined with the complexity of food matrices, restrict the applicability of UV detection for their accurate quantification. To address this challenge, coupling UV spectroscopy with liquid chromatography (LC) offers a robust analytical strategy by enabling the separation of small-molecule α-DCs from interfering matrix components prior to detection. Since small-molecule α-DCs are highly water-soluble and structurally lack chromophores, derivatization is often required to enhance their detectability and quantifiability by higher-sensitivity detection techniques. The frequently employed derivatizing reagents are *o*-phenylenediamine (OPD) [38], 4-nitro-OPD [69], 3,3′-diaminobenzidine (DAB) [83], and 2,4-dinitrophenylhydrazine (2,4-DNPH) [88] for high-performance liquid chromatography–ultraviolet detector (HPLC-UV) analysis. For instance, Lee et al. [64] used OPD as a derivatizing reagent and were the first to find small-molecule α-DCs in red ginseng products. Subsequently, Yang et al. [5] established and evaluated an HPLC-UV method based on 4-nitro-OPD as a derivatizing reagent and used it to measure the contents of GO, MGO, and DA in 35 Chinese herbs. The results showed the wide presence of small-molecule α-DCs in Chinese herbs, with concentrations generally comparable to those in various foods.

The principle of the derivatization reactions mentioned above is that the adjacent amino groups of derivatization reagents can react with the carbonyls of α-DCs to form quinoxaline structures. This process can markedly reduce the hydrophilicity of small-molecule α-DCs and substantially improve their sensitivity by HPLC-UV analysis. However, the derivatizing reactions by these reagents must be carried out at high temperatures for extended periods. Prolonged heating can promote the auto-oxidation of precursors in foods, leading to the formation of small-molecule α-DCs. This consequently raises the contents of small-molecule α-DCs, thereby compromising analytical accuracy. To resolve this problem, researchers [58] discovered that 3,3′-diaminobenzidine (DMN) and its derivative 4-(2,3-dimethyl-6-quinoxalinyl)-1,2-benzenediamine (DQB) could rapidly react with DA within 5 min at ambient temperature. On this basis, they developed two pre-column derivatization HPLC-UV approaches for quantifying DA concentrations in liquor and beer, respectively. Moreover, they subsequently adapted the DQB-based method to achieve simultaneous quantification of GO, MGO, and DA across diverse food matrices [59]. This method demonstrated low limits of detection (LODs) ranging from 0.02 μM to 0.05 μM and high recoveries from 85% to 103%. Compared with reported derivatizing procedures using other reagents, this method offers the key benefits of rapid derivatization at room temperature.

HPLC-UV not only improves the sensitivity and selectivity for quantifying small-molecule α-DCs but also mitigates UV absorption interference from complex and varied food matrices. However, this technique requires extra steps like derivatization. Both derivatization and separation processes are time-consuming, limiting their use for real-time monitoring of small-molecule α-DCs during food processing. Therefore, developing new derivatization agents to enhance selectivity and shorten reaction times, along with establishing standardized sample treatment protocols, is crucial for advancing HPLC-UV-based analysis of small-molecule α-DCs in complex food matrices.

#### 5.1.2. Fluorescence Spectroscopy

Fluorescence spectroscopy is another prominently employed technique for analyzing small-molecule α-DCs. Its working principle is photoluminescence. When a compound absorbs photons at a specific wavelength, it is excited to a higher energy state. As the compound returns to its ground state, it emits energy as fluorescence, characterized by a wavelength shift relative to the excitation source. By measuring the intensity and wavelength distribution of the resulting fluorescence, target compounds can be analyzed both qualitatively and quantitatively. However, owing to their high reactivity, instability, and lack of intrinsic fluorophores, the direct detection of small-molecule α-DCs by fluorescence spectroscopy is a challenge. Therefore, diverse techniques are employed for the fluorescence spectroscopy detection of small-molecule α-DCs, which can be classified into two main types: derivative reaction-based and probe-based methods.

(1) Derivative reaction-based fluorescence detection.

Table 2 summarizes the most widely employed derivatizing reagents for the fluorescence-based detection of small-molecule α-DCs. In 1994, Yamaguchi et al. [57] first developed an HPLC-fluorescence detector (HPLC-FLD) for the simultaneous quantification of GO, MGO, and DA in fermented foods. These small-molecule α-DCs were converted into their corresponding fluorescent derivatives by reaction with 1,2-diamino-4,5-methylenedioxybenzene (DMB). The LODs were 1.16–1.38 μM. To improve detection sensitivity, Rodríguez-Cáceres et al. [60] developed HPLC-FLD to quantify GO and MGO in wine without sample clean-up, using 3,4-diaminopyridine (3,4-DAP) as a novel derivatization reagent. The separation process was achieved within just 4 min, and the sensitivity was improved with LODs of 0.02 μM for GO and 0.006 μM for MGO. However, the derivatization process took 2 h. To resolve the problem, they [89] further developed a novel dispersive liquid–liquid microextraction (DLLME) method coupled with HPLC-FLD for the determination of GO, with the entire process completed in just a few minutes.

Owing to the high chemical reactivity and trace concentrations of small-molecule α-DCs in complex food matrices, rigorous sample preparation and enrichment are required to improve their detection sensitivity and analytical accuracy. However, conventional sample preparation approaches usually perform derivatization and extraction as separate steps, leading to laborious processes and extended processing durations. Furthermore, excessive sample transfer procedures elevate the risk of experimental errors, which may compromise the reliability of analytical outcomes. The integration of simultaneous derivatization and extraction can therefore significantly simplify the overall sample preparation procedures. Luo et al. [62] established a one-pot strategy for the detection of small-molecule α-DCs in beverages using HPLC-FLD. This method can achieve simultaneous derivatization and extraction/enrichment by combining a sample, 2,3-diaminonaphthalene as a derivative reagent, and magnetic adsorbent Fe_3_O_4_/MWCNTs-OH in a sample vial. The use of a magnetic adsorbent facilitates convenient phase separation through an external magnetic field, thereby simplifying and accelerating the entire sample preparation process. The LODs were as low as 0.4 nM for DA and 0.8 nM for GO and MGO. The aforementioned derivatizing agents, which are based on α-diamine reagents, may react with any organic molecule containing at least one aldehyde group, and they are not selective for small-molecule α-DCs. EI-Maghrabey et al. [84] recently developed a novel fluorogenic derivatization reaction in which GO underwent trimerization with ammonium acetate at 75 °C to form fluorescent 2,2′-biimidazole. This unique fluorogenic reaction exhibits excellent selectivity toward GO, with no reaction detected toward structurally analogous molecules such as malondialdehyde. Therefore, more highly selective and reactive derivatizing reagents specifically for small-molecule α-DCs can be designed and developed in the future.

(2) Probe-based fluorescence detection.

Fluorescent probes are a class of chemical or biological molecules that can specifically and selectively recognize or label target compounds, thereby inducing changes in their fluorescent properties. When target compounds are excited by an external light source, the attached fluorescence probe emits a signal at a distinct wavelength. By analyzing the intensity, wavelength, and lifetime of this fluorescence signal, both qualitative and quantitative identification of the target molecule can be achieved. With the advantages of real-time observability, robust specificity, and non-invasiveness, this technology has been found to have broad applications in biological imaging and food analysis [90,91]. Jana et al. [92] developed 1,8-diaminonaphthalene (DAN) as an efficient “turn-on” probe for the determination of formaldehyde, GO and MGO, but this probe could not distinguish these compounds from each other. For further improvement, they [93] developed a highly water-soluble new advanced fluorescent probe, sodium 4,5-diaminonaphthalene-1-sulfonate, which can discriminate monocarbonyl formaldehyde and GO/MGO by modulating the intramolecular charge transfer (ICT) process.

Most of the previously reported probes targeting small-molecule α-DCs exhibited emission wavelengths of less than 600 nm [91,92,93], making them susceptible to interference. Wu et al. [94] synthesized SWJT-18, a highly selective fluorescent probe, for the detection and visualization of GO, exhibiting a characteristic emission wavelength of 646 nm. To improve sensitivity, they [95] modified the fluorescent probe and named it AATC, which exhibited a robust fluorescence response toward GO with high selectivity and sensitivity (0.25 μM) over other α-DCs in aqueous solution. Moreover, it demonstrated promising applications in real samples such as honey, cookies, and bread, with excellent recoveries from 98.12% to 100.88%. Due to their similar structure, these fluorescence probes cannot distinguish between GO, MGO, and DA. In the future, intelligent detection platforms integrating data processing algorithms and artificial intelligence may address this problem.

#### 5.1.3. Other Spectroscopic Techniques

Nuclear magnetic resonance (NMR) represents an analytical technique that utilizes the magnetic properties of atomic nuclei under strong magnetic fields to investigate molecular structures, dynamic processes, and physical environments. It stands as one of the most powerful tools in modern analytical chemistry for determining molecular structures. Donarski et al. [96] established an NMR-based method for rapid analysis of MGO in manuka honey, requiring neither sample derivatization nor chromatographic separation. The method quantified MGO based on the concentration of its hydrate form. However, MGO is also present as an oligomer, which can reduce the accuracy of NMR-based quantification analysis for MGO.

Surface-enhanced Raman scattering (SERS) spectroscopy, leveraging its unique molecular fingerprinting capability, has emerged as a premier tool for the rapid, ultra-sensitive identification of chemical and biological analytes in complex food systems. When a target analyte is adsorbed onto the surface of noble metal (e.g., gold (Au) or silver (Ag)) nanomaterials, its Raman signals can be amplified by 10^6^–10^15^ orders of magnitude due to the intense electromagnetic field near the metal surface. Xia et al. [14] reported a one-step, nucleophilic addition-based SERS strategy for rapid identification and detection of MGO in manuka honey, which used silver-coated gold nanoparticles (Au@Ag NPs) combined with 8-thioguanosine (8-TG) as a highly selective SERS probe. The SERS strategy was achieved by detecting Raman intensity changes at 631 cm^−1^ and 700 cm^−1^ of N_2_-(1-carboxyethyl)-thioguanosine (CETG), and CETG was generated from the reaction between 8-TG and MGO on the Au@Ag NP surface. Notably, the Raman signal of CETG at 631 cm^−1^ increased, whereas that at 700 cm^−1^ decreased. The opposing intensity variation not only enabled highly selective MGO detection but also established an internal calibration mechanism, thereby minimizing interference from matrix or non-specific compounds during quantitative analysis of MGO. Furthermore, the entire analysis using this method can be completed within 20 min, representing a significant improvement in analysis speed compared with the conventional HPLC-FLD methods.

**Figure 4:**
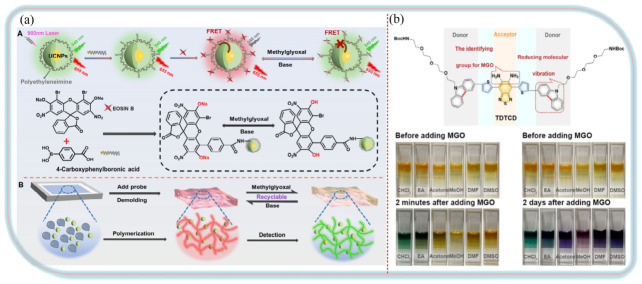
(**a**) NIR-excitable reversible ratiometric fluorescent hydrogel sensor for MGO detection (A: the formation process of the up-conversion nanoprobe and the reaction principle between the probe and MGO; B: the formation process of the hydrogel and the loading process of the probe in the hydrogel). This figure was adapted from ref. [97], with permission from the American Chemical Society, copyright 2026. (**b**) Molecular structure of the MGO-activated NIR-II probe TDTCD, along with optical images showing its reaction with MGO in various solvents. This figure was adapted from ref. [31], with permission from the American Chemical Society, copyright 2026.

Short-wavelength fluorescence detection often suffers from significant background interference, which can obscure the signals of small-molecule α-DCs and compromise the accuracy of quantitative analysis. In contrast, fluorescence probes operating in the near-infrared (NIR) region (800–1700 nm) offer a promising solution by eliminating background interference and enhancing photostability, thereby enabling reliable detection of small-molecule α-DCs in complex food matrices [31,97] (Figure 4a,b). For example, Kang et al. [97] reported a reversible ratiometric fluorescent hydrogel sensor excitable by NIR light (980 nm), in which near-infrared-activated up-conversion nanoparticles act as energy donors, while Eosin B functions as an energy acceptor, collectively constructing an integrated ratiometric nanophotonic probe. This configuration ensured detection accuracy by resisting diverse background fluorescence interferences in complex matrices and also enabled real-time monitoring of changes in MGO content. After MGO addition, the G/R ratio of the sensing film exhibited noticeable variation, enabling real-time quantitative determination of MGO in wines and honey with an LOD as low as 59 nM, demonstrating practical application in quality control and safety monitoring during food production.

### 5.2. Mass Spectrometry

Mass spectrometry (MS) is an effective strategy that ionizes sample molecules and enables precise qualitative and quantitative detection of target analytes based on their mass-to-charge ratios (*m*/*z*) and ionization mechanisms [98]. Target compound molecules are ionized at the ionization source using methods such as high-speed electron beams, strong electric fields, or lasers. The small-molecule α-DCs that cannot be directly detected by UV or FL would produce strong signals in the mass spectrum, with fast detection speed and high sensitivity. However, isomer differentiation and matrix effects remain critical challenges for MS-based analysis of small-molecule α-DCs. A viable solution to these issues is integrating separation techniques (e.g., chromatography, multi-capillary columns) with MS. Therefore, the most common MS methods include chromatography–mass spectrometry, mass spectrometry probes, and recently developed novel mass spectrometry techniques.

#### 5.2.1. Chromatography–Mass Spectrometry

Chromatography, including gas chromatography (GC) and liquid chromatography (LC), is a powerful separation technique that resolves small-molecule α-DCs and other compounds based on their differential distribution coefficients between the mobile and stationary phases. Owing to the characteristics of low molecular weight, high reactivity, and strong polarity of small-molecule α-DCs, chromatography–mass spectrometry detection may not provide sufficient sensitivity because of their retention on the column and thermal decomposition at elevated temperatures. For chromatography–mass spectrometry, modification to their structures by derivatization is essential to improve the chromatographic separation of small-molecule α-DCs, as well as their thermal stability and detection sensitivity. Derivatizing reagents are selected based on their compatibility with detector responses and the volatility of the resulting derivatives for GC-MS or LC-MS analysis. As shown in Table 2, we summarized common derivative reagents for small-molecule α-DCs detection with GC-MS or LC-MS.

(1) GC-MS.

GC-MS is highly suited for characterizing volatile, thermally stable analytes with molecular weights of no more than 1000 Da. To ensure that small-molecule α-DCs exhibit adequate stability and volatility for GC-MS analysis, derivatization of these compounds is required. The frequently employed derivatizing agents are *O*-(2,3,4,5,6-pentafluorobenzyl) hydroxylamine hydrochloride (PFBHA) [61], 2,2,2-trifluoroethylhydrazine (TFEH) [67,99], OPD [49,64], 5,6-diamino-2,4-hydroxypyrimidine, and 2,4,5-triamine-6-hydroxypyrimidine [10]. GC-MS coupled with headspace solid-phase microextraction (HS-SPME) constitutes a favored analytical approach for determining volatile components in diverse food matrices, enabling simultaneous extraction and pre-concentration of target analytes. Moreira et al. [61] reported a method based on HS-SPME coupled to GC-MS to quantify small-molecule α-DCs in different port wines; this method derivatized GO, MGO, and DA with PFBHA for 10 min and subsequently extracted for 30 min. The extraction of small-molecule α-DCs by HS-SPME was time-consuming. Recently, HS-SPME coupled with GC-MS has been optimized for the simultaneous measurement of seven carbonyl and dicarbonyl compounds, including GO, MGO, and DA, in infant formula [67]. This method decreased the extraction time to 10 min by elevating the temperature to 60 °C. To further simplify the pretreatment process and improve the extraction efficiency of small-molecule α-DCs, Lim et al. [99] developed a method involving simultaneous derivatization using TFEH and HS-SPME, with a total operation time of only 20 min. This method achieved the simultaneous determination of GO and MGO in alcoholic drinks and fermented foods via GC-MS, which had low quantification limits of 3.6 and 2.1 μg kg^−1^ for GO and MGO, respectively. The principle of HS-SPME involves exposing a fiber head coated with an adsorbent layer to the headspace vapor of a sample. Target analytes volatilize from the sample matrix and are adsorbed onto the fiber head for extraction. This technique offers simple operation without complex sample pretreatment. However, the fiber heads used are costly and prone to damage, and their extraction efficiency for low-volatility compounds is relatively low.

Given the critical role of small-molecule α-DCs in food safety and quality assessment, numerous sample preparation methods have been developed to enable their accurate quantification in complex food matrices. Santos et al. [100] introduce a fan-assisted air circulation-based extraction and pre-concentration technique for the detection of MGO, DA, and other compounds. The method significantly enhances analyte mass transfer kinetics and demonstrates high reproducibility. Dispersive liquid–liquid microextraction (DLLME), another sample preparation strategy, employs a mixture of dispersive and extraction solvents to extract target analytes from diverse food matrices, with the merits of low cost and low volatility requirements. Lee et al. [64] applied DLLME combined with GC-MS to measure GO, MGO, and DA in red ginseng products. The proposed method employs 100 μL of chloroform as the extraction solvent, 200 μL of methanol as the disperser solvent and 0.5 g of OPD as the derivatization reagent, achieving high sensitivity with LODs of 1.30 μg kg^−1^ to 1.86 μg kg^−1^. Recently, based on DLLME GC-MS, they further optimized the volume of dispersing solvents (1 mL of methanol) and derivatization reagents (500 μg of OPD) for the determination of α-DCs in sesame oils, with LODs as low as 0.43 μg kg^−1^ for GO, 0.22 μg kg^−1^ for MGO, and 0.86 μg kg^−1^ for DA [49]. The differences in the dispersion solvent volume and the derivatization reagent dosage between the two studies suggest that the optimal conditions for the determination of small-molecule α-DCs by DLLME are matrix-dependent for different samples.

DLLME is widely used for its rapidity, simplicity, high enrichment factor, and extraction efficiency. Nevertheless, conventional DLLME procedures rely heavily on hazardous solvents, such as chloroform (CHCl_3_) and dichloromethane (CH_2_Cl_2_). In response to growing environmental and safety concerns, research has prioritized the development of greener DLLME approaches. Green DLLME techniques typically employ less deleterious extraction solvents, including ionic liquids [101] and low-density solvents [100,102]. These modifications are integrated with streamlined sample preparation workflows, which collectively reduce the total analysis time and minimize reagent usage [103]. Silva et al. [100] developed a greener analytical strategy involving ultrasound-assisted DLLME (UA-DLLME), which enabled a one-step simultaneous extraction and derivatization of target carbonyl compounds in coffee samples before GC-MS analysis. In their research, the conventional solvents employed in DLLME procedures were replaced with 1-octanol and isooctane as the extraction and dispersive solvents, respectively, which offered the advantages of low density and minimal toxicity. Meanwhile, the volume of extraction solvent was reduced to 60 μL. This proposed method demonstrated excellent analytical performance for the simultaneous measurement of nine carbonyl compounds, including GO, MGO, and DA.

(2) LC-MS.

LC-MS is another preferred technique for analyzing highly polar, non-volatile, thermally unstable, and macromolecular compounds. The analytical principle of LC-MS is similar to that of GC-MS, with the difference being that both the mobile phase and sample at the outlet of the LC system are in liquid form. Therefore, the liquid target component in the solvent must be efficiently converted into gaseous ions suitable for mass spectrometry analysis. This process is fundamentally governed by the ion source technology. The commonly used ionization technologies are electrospray ionization (ESI) and atmospheric pressure chemical ionization (APCI). Like GC-MS, LC-MS detection of small-molecule α-DCs also requires derivatization. The commonly used derivatizing reagents are OPD [55,56,63] and 2,4,5-triamine-6-hydroxypyrimidine (TRI) [10]. Hurtado-Sánchez [10] first reported the quantification of α-DCs using an ultra-HPLC (UHPLC) method with ESI-MS. This research assessed the commonly employed derivatizing reagents OPD, TRI, and 5,6-diamino-2,4-dihydroxypyrimidine (DDP) under their optimal reaction conditions. The results demonstrated that TRI was the optimal derivatization reagent for quantifying target compounds in sugar-rich food samples. This method was successfully validated for the quantification of GO, MGO, DA, and other α-DCs in diverse honey samples. Based on HPLC-ESI-MS, Song et al. [55] reported a unified UHPLC-ESI-MS method for the detection of α-DCs in propolis. In their study, propolis extracts were collected and cleaned on an HLB solid-phase extraction column to remove lipids and beewaxes, followed by derivatization with OPD to form α-dicarbonyl quinoxaline compounds (α-DC-Qs). This pretreatment protocol effectively eliminated matrix interferences, enabling high-sensitivity detection of α-DCs in propolis through subsequent instrumental analysis. ESI-MS conditions were established to achieve accurate quantification of 10 α-DC-Qs, including methylglyoxal quinoxaline (MGO-Q) and diacetyl quinoxaline (DA-Q), by monitoring characteristic precursor ions [M·H]^+^ (M represents MGO-Q or DA-Q) and fragment ions within the complex propolis extracts.

Drift-time ion mobility spectrometry (IMS) is a relatively novel technique that separates ions based on their structural and conformational characteristics, offering distinct advantages in enhancing the sensitivity and selectivity required to distinguish α-DC isomers [104,105]. LC-IMS-MS generates four-dimensional analytical data, integrating retention time (from LC), collision cross-section (from IMS), mass-to-charge ratio (*m*/*z*, from MS), and ion intensity. The collision cross-section value, derived from drift time, correlates strongly with the inherent physicochemical characteristics of compounds, such as charge, molecular weight, and structural conformation. This multidimensional dataset enables the separation of target analytes based on physicochemical properties, significantly enhancing the resolution of isomers and the identification of small-molecule α-DCs in complex food matrices. Yan et al. [104] developed UHPLC-IMS-MS/MS, integrated with multivariate statistical analysis to discriminate the adulteration of acacia honey with high-fructose corn syrup. The method relies on the quantitative profiling of α-DCs, key biomarkers of honey authenticity. Currently, the applications of IMS-MS for small-molecule α-DC detection and analysis are still in the early stages. Owing to its great potential for isomer separation, IMS-MS-based analytical strategies warrant further development and optimization.

#### 5.2.2. Mass Spectrometry Probe Technique

Most existing derivatizing reagents react with small-molecule α-DCs to form quinoxaline structures, which require harsh reaction conditions and complex derivatization processes. Furthermore, these reagents exhibit limited selectivity toward small-molecule α-DCs, leading to potential interference from co-existing compounds. For example, some reagents, such as OPD, 4-nitro-OPD, and 4-methoxy-OPD, require reaction temperatures above 60 °C, which can oxidize the precursors of small-molecule α-DCs and decompose derivatization products, leading to inaccurate quantification of small-molecule α-DC levels. Meanwhile, other derivatization reagents, such as 4-methoxy-OPD [85], exhibit reactivity at low temperatures, yet their practical utility is constrained by prolonged reaction duration (several hours), which affects analytical reliability and efficiency. Accordingly, the development of highly sensitive and selective derivatization reagents is essential for accurate MS-based analysis of small-molecule α-DCs. Qi et al. [106] synthesized a novel high-sensitivity mass spectrometry probe, 3-benzyl-2-oxo-4λ^3^-thiazolidine-4-carbohydrazide (BOTC), which was functionalized with hydrazyl groups for the targeted recognition of carbonyls. Coupled with UHPLC-MS/MS, this method enabled the simultaneous separation and quantification of GO, MGO and DA in red wine, demonstrating excellent sensitivity, with LODs as low as 12.5–50 fmol, high accuracy, with intra- and inter-day precision variations between 0.1% and 5.7%, and efficiency, with recoveries of 103.3–110.2%. Based on this study, Han et al. [7] also applied a novel BOTC MS probe with UHPLC-MS/MS for the simultaneous determination of GO, MGO, and DA, with high sensitivity in several alcoholic beverages.

In summary, although GC-MS and LC-MS offer high analytical accuracy and robust resistance to matrix interferences via chromatographic separation, they are often criticized for complex sample pretreatment and derivatization, cumbersome operational procedures, and slow detection speed. These limitations make them difficult to use in the real-time monitoring of small-molecule α-DCs during food processing.

#### 5.2.3. MS-Based Novel Techniques

**Figure 5:**
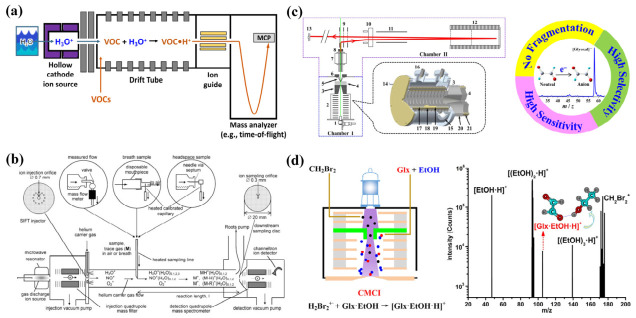
Structures and ionization mechanisms of PTR-MS (**a**), figure adapted from ref. [110], with permission from John Wiley and Sons, copyright 2026, and SIFT-MS (**b**), figure adapted from ref. [111], with permission from John Wiley and Sons, copyright 2026. The structure of electron attachment reaction ionization mass spectrometry and mass spectra of GO with characteristic ions of [C_2_H_2_O_2_]^−^, (**c**) figures adapted from ref. [33], with permission from the American Chemical Society, copyright 2026, and ref. [35], with permission from Peking University, copyright 2026 (1, laser ablation ion source; 2, drift tube; 3, ion funnel; 4, stacked ring radio frequency ion guide; 5, skimmer (2 mm diameter); 6, 8, and 11, Einzel lenses; 7, linear ion trap; 9, electrodes for accelerating ions; 10, deflectors; 12, reflector; 13, dual microchannel plate detector; 14, port for drift gas; 15, port for capacitance manometer; 16, electrical feedthrough; 17, stainless steel screw; 18 and 21, polyetheretherketone spacer; 19, polyetheretherketone tube; 20, polytetrafluoroethylene washer). Diagram of a cluster-mediated CH_2_Br_2_^+^ chemical ionization (CMCI) source and mass spectra of GO with characteristic ions of [C_2_H_2_O_2_·EtOH·H]^+^ (**d**), figure adapted from ref. [34], with permission from Elsevier, copyright 2026.

Soft ionization mass spectrometry is another crucial and versatile technique for the real-time measurement of trace organic compounds, including small-molecule α-DCs, in various fields [34,107]. Soft ionization is a low-energy ionization technique that enables sample molecules to be ionized by absorbing as little energy as possible, which generates gas-phase molecular ions with minimal or no chemical bond breaking. Soft ionization mass spectrometry exhibits distinct advantages for small-molecule α-DC analysis in foods, including rapid detection, high sensitivity, high molecular ion yield, and simple mass spectra interpretation. The widely used soft ionization techniques include chemical ionization (CI) and photoionization (PI). Common CI mass spectrometry techniques include proton transfer reaction mass spectrometry (PTR-MS) and selected ion flow tube mass spectrometry (SIFT-MS), which employ H_3_O^+^, NO^+^, or O_2_^+^ as the primary reagent ions. As shown in Figure 5a,b, PTR-MS employs H_3_O^+^ generated from H_2_O as reagent ions. Its ionization mechanism involves a mild proton transfer reaction between target organic molecules and H_3_O^+^, which facilitates the formation of protonated molecular ions with minimal fragmentation. This proton transfer reaction is governed by the relative proton affinity (PA) of the analyte and H_2_O. Specifically, target molecules must exhibit a PA exceeding that of water (PA value of 690 KJ/mol at 298 K) to undergo efficient protonation. Besides H_3_O^+^, the common reagent ions of SIMT-MS employed in the determination of analytes also contain O_2_^+^ (from O_2_) and NO^+^ (from NO or N_2_ and O_2_) reagent ions [108,109]. O_2_^+^ cations facilitate charge transfer reactions with analytes to produce molecular ions M^+^, whereas NO^+^ drives hydride abstraction (yielding [M−H]^+^) or association reactions (forming [M·NO]^+^ adducts). These pathways enable selective detection of diverse analyte classes by tailoring reagent ion selection to target compounds.

PTR-MS or SIFT-MS has also been used to analyze small-molecule α-DCs, especially GO and MGO. However, some issues remain. Several studies [112,113,114] concluded that the quantification of GO and MGO with PTR-MS exhibited relatively low sensitivity, especially under high-humidity conditions. This limitation arises from the comparable or lower PA of GO and MGO relative to water. The reaction between O_2_^+^ reagent ions and GO by charge transfer results in the molecule ion GO^+^, but also in at least one fragment ion. A prominent fragment ion observed in GO analysis is HCHO^+^ (*m*/*z* 30). Notably, this ion can also be generated through charge transfer reactions between O_2_^+^ reagent ions and formaldehyde, creating an interference effect that complicates accurate quantification. The reaction of NO^+^ reagent ions with GO is only an association to generate [M·NO]^+^. However, sensitivity is significantly influenced by humidity. Under dry conditions, the LOD of GO reaches ppbv (parts per million by volume) levels, whereas elevated humidity significantly reduces sensitivity [115]. Consequently, there is an urgent demand for efficient MS analytical methods capable of rapidly determining small-molecule α-DCs. Lu et al. [33] developed an electron attachment reaction ionization MS for quantifying GO with high sensitivity. Shown in Figure 5c, parent anions (GO^−^) were generated without fragmentation, and the LOD reached as low as 52 pptv (parts per trillion by volume) in just one minute.

PI-MS represents another attractive soft ionization strategy that offers the distinct advantages of high sensitivity, high molecular ion yields, and facile spectrum interpretation [116,117,118,119,120]. The employment of low-cost and compact discharge lamps, such as a krypton (Kr) lamp, as the vacuum ultraviolet (VUV) light source has expanded the applicability of PI-MS for on-line and real-time analysis of trace organic species [116,118]. Nevertheless, conventional VUV Kr lamp-based PI-MS suffers from limited sensitivity and narrow analyte coverage, primarily due to the low photon flux and suboptimal ionization energy of the light source. This hinders the sensitive detection of small-molecule α-DCs, which typically exhibit low photoionization cross-sections and high ionization energies. As the ionization source pressure increases, ion–molecule reactions become increasingly prevalent within the source. These reactions can act as another ionization pathway for analyte molecules [34,117]. As shown in Figure 5d, Wan et al. [34] reported a VUV-Kr lamp-based, cluster-mediated CH_2_Br_2_^+^ chemical ionization MS for GO analysis. In their study, the use of photoionization-generated CH_2_Br_2_^+^ as the reagent ion and co-sampling of GO with high-concentration ethanol (EtOH) promoted ion–molecule reactions between CH_2_Br_2_^+^ and glyoxal-ethanol dimers. This process significantly enhanced the ionization efficiency of GO, resulting in the formation of the protonated cluster ion [GO·EtOH·H]^+^. When coupled with time-of-flight MS, the analytical capacity of this system was validated through the trace quantification of GO in food contact papers within 30 s, providing new insights into soft ionization MS for organic compound determination, especially compounds with high ionization energies and low ionization cross-sections.

Currently, the research and applications of direct soft ionization MS for small-molecule α-DCs are relatively infrequent. Future research should prioritize the development of novel, matrix-tolerant soft ionization MS techniques to address two critical limitations in small-molecule α-DC analysis: (1) expanding detection capabilities beyond GO to MGO and DA and (2) enabling simultaneous quantification of multiple α-DCs in complex samples. Such advancements would reduce reliance on labor-intensive separation methods and support real-time analysis of small-molecule α-DCs in foods.

## 6. Limitations and Prospects

Small-molecule α-DCs are found in various foods and beverages; notably, they are also widely present in Chinese herbal medicines. Their levels vary greatly among different foods. The main factors affecting their occurrence include the type and content of sugar, ingredients, moisture content, food processing methods, and storage conditions. Due to their highly toxic and chemically reactive nature, a large number of studies have indicated that excessive exposure to small-molecule α-DCs can accelerate the onset and development of numerous metabolic diseases. However, existing available toxicological evidence of small-molecule α-DCs mainly originates from short-term, high-dose in vitro and animal model investigations. In contrast, the human toxicokinetic behaviors and long-term chronic toxicological characteristics of these small-molecule α-DCs remain virtually unexplored. Meanwhile, quantitative criteria and standardized protocols are not yet established to evaluate risks associated with multi-pathway co-exposure. Additionally, the interactive mechanisms governing the formation and conversion of small-molecule α-DCs and the causal relationships between continuous low-dose exposure and human chronic disorders lack robust epidemiological evidence for validation. To address the above deficiencies, the development of highly sensitive and selective analytical techniques for small-molecule α-DCs is critical.

Several analytical techniques have been developed for the qualitative and quantitative analysis of small-molecule α-DCs. As shown in Table 3, current detection techniques face a few challenges: (1) Complex pretreatment and derivatization. Due to high polarity, instability, and lack of strong chromophores in small-molecule α-DCs, derivatization is necessary. This requires stringent conditions or prolonged time that can lead to the formation of side products or the decomposition of small-molecule α-DCs, thereby interfering with their accurate quantification. (2) Poor accuracy and sensitivity of analytical methods. The presence of structural isomers (e.g., malonaldehyde and MGO) and complex food matrices compromises the accurate measurement of small-molecule α-DCs. For example, PI MS is highly sensitive to humidity. Specifically, sensitivity declines as sample humidity increases. (3) Challenges in the simultaneous detection of multiple small-molecule α-DCs. Fluorescent probe-based detection has emerged as a promising rapid screening tool. However, it is characterized by tedious probe synthesis and high specificity. Therefore, it is difficult to use for the simultaneous detection of multiple small-molecule α-DCs.

Future research should develop rapid and specific derivatization reagents to simplify sample pretreatment and realize rapid analysis of small-molecule α-DCs. The molecular imprinting technique can specifically and selectively recognize template molecules from complex matrices by a “lock-and-key” mechanism. When using one of the small-molecule α-DCs as the template molecule, synthesizing molecular imprinting polymers holds promise for highly specific and selective extraction of small-molecule α-DCs from complex and varied food matrices. In addition, using soft ionization mass spectrometry, modifying the structures of small-molecule α-DCs or developing novel ionization techniques could provide new insights for highly sensitive, strongly selective, and rapid simultaneous analysis of multiple small-molecule α-DCs. Moreover, the use of isotopically labeled internal standards and harmonized sample preparation protocols also improves the accuracy and precision of small-molecule α-DC analysis. Based on this, future research will also focus on elucidating the formation and interconversion pathways, filling gaps in human toxicokinetic profiles encompassing absorption, distribution, metabolism, and excretion (ADME), unraveling toxicological mechanisms triggered by prolonged low-dose exposure, and refining targeted risk assessment systems for susceptible populations exposed to small-molecule α-DCs.

## 7. Conclusions

GO, MGO, and DA, classified as small-molecule α-DCs, are produced mainly through the Maillard reaction, caramelization, lipid peroxidation, and enzymatic reactions during the heating and storage of foods. People are continuously exposed to small-molecule α-DCs through their diets, whose severe toxicity and high chemical reactivity have a negative influence on human health. Moreover, small-molecule α-DCs can also act as important intermediates and precursors by inducing other toxic compounds. Small-molecule α-DCs and their derived hazards can accelerate the onset and progression of multiple metabolic diseases. However, their pathological processes, toxicological data, and exposure assessments remain poorly elucidated. To address these issues, quantifying these small-molecule α-DCs in various foods is crucial. Therefore, this paper reviewed detection techniques for standardizing the food production process and reducing the health hazards associated with small-molecule α-DCs and highlighted recent advances in the formation mechanisms, occurrence, and toxicity of small-molecule α-DCs. This work provides recommendations for the quantitative detection of trace small-molecule α-DCs. Chromatography and chromatography–mass spectrometry are still frequently used techniques for the quantitative determination of small-molecule α-DCs. Due to their polarity, trace content in complex foods, and inability to absorb ultraviolet light, sample pretreatment is necessary yet time-consuming. Although several rapid detection techniques have emerged in recent years, including mass spectrometry probes and soft ionization mass spectrometry, the development of rapid, eco-friendly, highly sensitive, and accurate analytical methods, such as the use of isotopically labeled internal standards, the development of novel ionization techniques and harmonized sample preparation protocols, remains a priority for future research.

## Figures and Tables

**Table 1:** Contents of small-molecule α-DCs in foods (mg/kg or mg/L).

Foodstuffs	Specification	GO	MGO	DA	Geographical Origin	References
Sauces	Soy sauce	5.6–31.7	14.4–116	0.5–5.5	Republic of Korea	Kim et al. [8]
		11.8	5.0	-	The Netherlands	Maasen et al. [1]
	Vinegar	0.01–5.7	0.04–12	-	The Netherlands	Maasen et al. [1]
	Tomato sauce	3.4–6.9	1.3–3.0	-		
	Jams	1.84–6.16	0.63–29.78		Turkey	Çatak et al. [13]
Oils	Fish oil	2.2–12.4	0.2–4.5	n.d.	USA	Suh et al. [48]
	Olive oil	0.03	0.04	-	The Netherlands	Maasen et al. [1]
	Sesame oils	0–0.18	0–0.99	0–0.22	Republic of Korea	Lee et al. [49]
Turkish traditional foods	Turkish delight	3.2–18.4	0.3–5.2	-	Turkey	UĞUR et al. [50]
	Baklava	3.4–8.2	0.2–3.6	-		
Bread	Bread	1.5–11	2.3–26	-	The Netherlands	Maasen et al. [1]
	Bread condiments	2.4–37	1.7–21	-		
Breakfast cereals	Wheat and corn, chocolate	0.1–15.8	1.1–12.0	-	Turkey	Cengiz et al. [51]
	Cereal, rice, and fruits	0.08–1.06	2.22–4.46	-		
	Cornflakes	0.73	0.73	-	The Netherlands	Maasen et al. [1]
Snacks	Cookies	4.8–20.5	3.7–78	-	Spain	Arribas-Lorenzo et al. [52]
	Chips	0.9–14.6	1.2–6.6	-	Turkey	Cengiz et al. [51]
	Crackers	3.4–19.4	7.3–14.0	-		
	Chocolate	0–2.28	0–3.97	-	Turkey	Ede-Cintesun et al. [6]
	Dried fruits	0.2–48.7	1.4–254.1	0.5–18.4	Turkey	Aktağ et al. [53]
Honey	Acacia honey	0.6–3.2	0.8–4.8	0.7–2.4	China	Yan et al. [9]
	Manuka honey	9.2–17	2.5–736	-	The Netherlands	Maasen et al. [1]
		-	124.5–530	-	China	Xia et al. [14]
	Italian honey	-	0.4–24.1	-	Italy	Terio et al. [54]
	Propolis	-	2.34–44.94	0.89–12.36	China	Song et al. [55]
Dairy	Milk and milk products	0–0.6	0–1.4	0–49.5	China	Zhang et al. [56]
	Yoghurt	0.15–0.92	0.5–1.27	0.9–2.19	Japan	Yamaguchi et al. [57]
	Cheese	0.03–0.35	0.12–0.96	-	The Netherlands	Maasen et al. [1]
	Eggs	0.15–0.32	0.11–0.57	-		
Beverages	Beer	1.29–6.87	1.44–8.41	n.d.–3.72	Republic of Korea	Jeong et al. [11]
		0.23–0.75	0.86–0.97	0.016–0.31	China	Wang et al. [58], Wang et al. [59]
		0.023–0.041	0.086–0.24	0.043–0.052	Japan	Yamaguchi et al. [57]
	Wine	0.4–3.0	0.5–1.8	-	Spain	Rodríguez-Cáceres et al. [60]
		0.25–10.12	0.51–5.41	0.97–2.4	Portugal	Moreira et al. [61]
		0.33–60.48	0.42–78.13	n.d.–55.06	Republic of Korea	Jeong et al. [11]
		1.1–8.9	0.21–3.21	0.29–8.8	Spain	Hurtado-Sánchez et al. [32]
	Juice	0.02–0.04	0.03–0.28	0.006–0.29	China	Luo et al. [62]
	Tea	0.02–5.2	0.02–5.69	0.08–0.8	China	Zhu et al. [63], Luo et al. [62], Wang et al. [59]
Meat	Flounder seafood condiments	0.39–18.27	1.84–367.13	0.09–2.50	China	He et al. [46]
	Pork/beef	1.0–6.2	1.7–3.9	-	The Netherlands	Maasen et al. [1]
	Fish	0.15–1.4	0.9–4.1	-		
	Chicken fillet	0.3–1.8	1.6–2.3	-		
Chinese herbal medicines	Red ginseng	0.19–4.27	1.33–4.8	0–0.83	Republic of Korea	Lee et al. [64]
	Fruit-based	0.84–6.0	1.11–28.95	1.8–18.75	China	Yang et al. [5]
	Root and rhizome-based	1.35–22.65	2.7–33.15	n.d.–2.85		
	Flower and leaf-based	n.d.–9.9	n.d.–55.5	n.d.–7.32		
	Seed-based	0.41–12.3	n.d.–14.4	n.d.–1.95		
Other foods	Coffee beans	11.2–23.7	19.95–82.82	10.36–23.72	China	Chen et al. [4]
	Coffee	0.81–7.74	12.1–89.08	0.43–6.74	Republic of Korea	Lee et al. [65], Park et al. [66]
	Infant formula	0–1.46	0.2–1.41	n.d.–0.89	Spain	Custodio-Mendoza et al. [67]

GO, glyoxal; MGO, methylglyoxal; DA, diacetyl; n.d., some samples were not detected in the cited original study; -, not involved in the original cited studies cited.

**Table 2:** Commonly used methods for small-molecule α-DC analysis by chromatographic techniques with different detectors.

Derivatizing Agent	Structure	Method	Matrix	Target α-DCs	LODs	References
*o*-phenylenediamine (OPD)	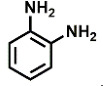	HPLC-UV	Honey	MGO	MGO (0.04 mg/kg)	Terio et al. [54]
4-nitro-1,2-phenylenediamine (NPDA)	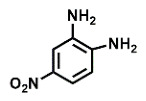	HPLC-UV	Chinese herbal medicines	GO, MGO and DA	GO (0.002 mg/L), MGO (0.002 mg/L) and DA (0.006 mg/L)	Yang et al. [5]
4-(2,3-dimethyl-6-quinoxalinyl)-1,2-benzenediamine (DQB)	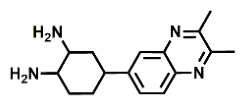	HPLC-UV	Fermented foods, carbonated drinks and coffee	GO, MGO and DA	GO (0.003 mg/L), MGO (0.004 mg/L) and DA (0.002 mg/L)	Wang et al. [59]
3,3′-diaminobenzidine (DAB)	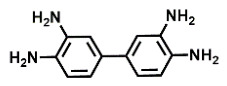	HPLC-UV	Liquors	DA	DA (0.008 mg/L)	Wang et al. [83]
1,2-diamino-4,5-methylenedioxybenzene (DMB)	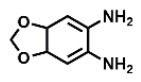	HPLC-FLD	Fermented foods	GO, MGO and DA	GO (0.015 mg/kg), MGO (0.019 mg/kg) and DA (0.034 mg/kg)	Yamaguchi et al. [57]
3,4-diaminopyridine (3,4-DAP)	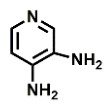	HPLC-FLD	Wines	GO and MGO	GO (0.001 mg/L), MGO (0.0004 mg/L)	Rodríguez-Cáceres et al. [60]
2,3-diaminonaphthalene (DMN)	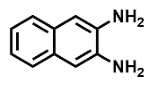	HPLC-FLD	Tea, coffee, fresh juices	GO, MGO and DA	GO (0.00005 mg/L), MGO (0.00006 mg/L) and DA (0.00003 mg/L)	Luo et al. [62]
2,3-diaminonaphthalene (DMN)	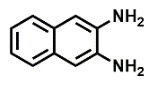	HPLC-FLD	Traditional Chinese medicines	GO, MGO and DA	GO (0.0002 mg/L), MGO (0.0002 mg/L) and DA (0.0002 mg/L)	Xu et al. [70]
ammonium acetate	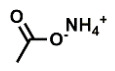	HPLC-FLD	Ethanol	GO	GO (0.0009 mg/L)	Mahmoud et al. [84]
4-methoxy-o-phenylenediamine (MPDA)	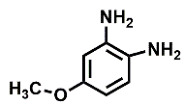	HPLC-FLD	High-fructose agave syrups	GO, MGO and DA	GO (0.07 mg/kg), MGO (0.06 mg/kg) and DA (0.05 mg/kg)	Escobosa et al. [85]
*O*-(2,3,4,5,6-pentafluorobenzyl)hydroxylamine hydrochloride (PFBHA)	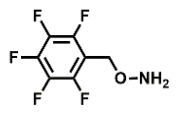	GC-MS	Port wines	GO, MGO and DA	GO (0.00006 mg/L), MGO (0.00004 mg/L) and DA (0.00007 mg/L)	Moreira et al. [61]
Pentafluorophenylhydrazine (PFPH)	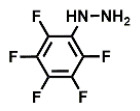	GC-MS	Infant formula	GO, MGO and DA	GO (0.015 mg/L), MGO (0.015 mg/L) and DA (0.016 mg/L)	Custodio-Mendoza et al. [67]
*o*-phenylenediamine (OPD)	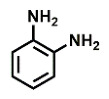	GC-MS	Red ginseng	GO, MGO and DA	GO (0.0013 mg/L), MGO (0.0019 mg/L) and DA (0.0015 mg/L)	Lee et al. [64]
2,4,5-triamine-6-hydroxy-pyrimidine (TRI)	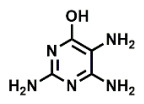	UHPLC-MS	Honey	GO, MGO and DA	GO (0.0074 mg/L), MGO (0.0072 mg/L) and DA (0.0083 mg/L)	Hurtado-Sánchez et al. [10]
*o*-phenylenediamine (OPD)	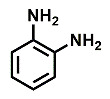	UHPLC-MS	Propolis	MGO and DA	MGO (0.073 mg/kg) and DA (0.025 mg/kg)	Song et al. [55]
3-benzyl-2-oxo-4λ^3^-thiazolidine-4-carbohydrazide (BOTC)	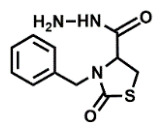	UHPLC-MS	Beer	GO, MGO and DA	GO (0.0015 mg/L), MGO (0.0022 mg/L) and DA (0.0011 mg/L)	Han et al. [7]

**Table 3:** Comparison of the performance of different analytical techniques.

Analytical Techniques	Advantages	Limitations	Sample Preparation	Sensitivity	Analysis Time	Applications
HPLC-UV	good separation capacity, simple operation, low instrument and running costs	target molecules with chromophores, derivatization required for non-UV-absorbing analytes, poor selectivity	solvent selection, derivatization	μg/g–mg/g	a dozen minutes to hours	detect GO, MGO and DA simultaneously in foods, including Chinese herbs, liquor, beer and honey
HPLC-FLD	better selectivity, relatively minor food matrix interference compared to HPLC-UV	only fluorescent compounds are detectable, needs pre- or post-column derivatization	derivatization of small-molecule α-DCs to generate fluorophores, light protection	ng/g–μg/g	several minutes to hours	detect GO, MGO and DA simultaneously in fermented foods, alcoholic beverages and drinks
Probe-based fluorescence spectroscopy	selectively recognize or label small-molecule α-DCs	complex probe synthesis, difficult to distinguish between GO, MGO and DA	probe synthesis, liquid sample preparation	μg/g	several minutes to several tens of minutes	biological imaging and food analysis, such as honey, cookies, and bread
GC-MS	good separation capacity, high sensitivity	cannot analyze non-volatile, thermally labile, or high-molecular-weight compounds, derivatization takes several minutes to hours	derivatization to improve volatility and reduce polarity of small-molecule α-DCs	ng/g	a dozen minutes to hours	two most commonly used analytical techniques for the simultaneous detection of GO, MGO and DA in drinks, fermented foods, meat, oils, milk, honey, Chinese herbs and other foods
LC-MS	wide analyte coverage: polar, thermolabile, and high-molecular-weight substances, multiple separation modes for diverse matrix samples	longer analysis time than GC-MS, derivatization takes several minutes to hours	particle-free, mobile-phase selection	pg/g–ng/g		
MS	ultra-high sensitivity, rapid, good qualitative specificity	expensive instrument purchase and maintenance, cannot separate mixtures independently	convert small-molecule α-DCs into gaseous molecules to facilitate ionization	fg/g–pg/g	within 1 min	GO in food contact papers
SERS	fast operando detection, simple pretreatment, portable	complex matrix interference	make samples easy to adsorb to Au/Ag substrates, reduce fluorescence background	ng/g	within 20 min	MGO in manuka honey
NMR	non-destructive, full structure elucidation	low sensitivity, expensive operation	full dissolution in deuterated solvent	mg/g	a dozen seconds	MGO in honey

## Data Availability

No new data were created or analyzed in this study. Data sharing is not applicable to this article.
